# Isolated injury, Charlson Comorbidity Index, and transfer from another facility are associated with delay in antibiotic administration: a retrospective study of 963 patients with open fractures

**DOI:** 10.1097/OI9.0000000000000300

**Published:** 2024-03-27

**Authors:** Tyler J. Pease, G. Wells Ducas, Michael L. Raffetto, Andrew C. Bernard, Jalen A. Martin, Paul E. Matuszewski

**Affiliations:** a Department of Orthopaedic Surgery and Sports Medicine, University of Kentucky College of Medicine, Lexington, KY; b Department of Orthopaedics, University of Maryland, Baltimore, MD; c Department of Surgery, University of Kentucky College of Medicine, Lexington, KY

**Keywords:** infection, open fracture, antibiotics

## Abstract

**Purpose:**

To identify factors associated with delays in administration and pharmacy and nursing preparation of antibiotics for patients with open fractures.

**Design:**

Retrospective review.

**Setting:**

Level I trauma center.

**Patients:**

Nine hundred sixty-three adults with open fractures administered antibiotics.

**Main Outcome Measurements:**

Delay in antibiotic administration greater than 66 minutes from arrival and significant pharmacy-related and nursing-related delay.

**Results:**

Isolated injury, Charlson Comorbidity Index, and transfer from another facility were associated with delay in antibiotic administration greater than 66 minutes. Injury Severity Score, transfer, and trauma team activation were associated with pharmacy-related or nursing-related delay.

**Conclusion:**

Interventions to reduce antibiotic administration time for open fractures should focus on early identification of open fractures and standardization of antibiotic protocols to ensure timely administration even in complex or resource-scarce care situations.

**Level of Evidence:**

Prognostic level III.

## 1. Introduction

Infections remain common despite the routine use of antibiotics in the early management of open fractures.^1–4^ Earlier antibiotic administration has been shown to correlate with lowered rates of infection in some fracture types, particularly in open tibia fractures.^5–9^ Reducing the time between injury and antibiotic administration is therefore likely to improve patient outcomes, with current guidelines for the treatment of open fractures agreeing that administering antibiotics “as soon as possible.”^10–13^ It is unknown what factors in patient care may contribute to delays in antibiotic administration. Delays in antibiotic administration may be due to prehospital factors including arrival of emergency medical services (EMS), extrication or transport, or in-hospital factors including triage, assessment, and administration of antibiotics. These factors are specific to the health system, EMS, hospital, patient, and injury. Identifying such factors may facilitate targeted, systematic interventions to reduce delays.

The purpose of this study was to determine which patient-specific and systemic factors are associated with a delay of antibiotic administration after a patient arrives at the hospital. We hypothesized that isolated injuries and patients with dorsally located open wounds would be associated with an increased risk of antibiotic delay because of decreased triage priority and the increased difficulty of identifying wounds located dorsally.

## 2. Materials and Methods

An IRB-approved, retrospective review of the medical record was conducted at a level 1 regional trauma center. We identified all patients older than 18 years treated with debridement and irrigation between January 1, 2013, and December 31, 2020, for an open fracture, using *Current Procedural Terminology* codes 11010, 11011, and 11012. Standard of care at our institution is to administer preselected, protocolized antibiotics as soon as an open fracture is identified (cephazolin or clindamycin for those with allergy, with the addition of gentamicin for contaminated and/or high energy injuries), apart from transferred patients who have documented antibiotic administration before arrival. Patients were excluded from analysis if clinical documentation noted the administration of antibiotics before arrival, either at a referring facility or by emergency medical services.

The primary outcome for this study was a delay of antibiotic administration, defined as greater than 66 minutes between the time from arrival to the first administration of appropriate antibiotics (either cefazolin or clindamycin). Previous studies have shown that 66 minutes of time elapsed from injury to antibiotic administration is associated with an increased rate of infection in severely injured open tibia fractures. This was selected to maximize the sensitivity of detecting modifiable risk factors for delay.^6,8^ Our secondary outcome was a pharmacy-related or nursing-related delay of antibiotic administration described as the interval of time between when an order is placed in the EMR and when the drug is documented as delivered. We defined a pharmacy or nursing delay as administration of antibiotics at a time point greater than 1 interquartile range above the median number of minutes between the time an order was placed for antibiotics and the time the antibiotic was administered (median 20.3 minutes; interquartile range 58.1 minutes; a difference greater than 78.4 minutes).

Demographic and injury characteristics were retrieved from the medical record and assessed as potential associated variables: age, sex (male/female), isolated injury (defined as injury to a single Abbreviated Injury Score [AIS] body region, with each extremity considered independently), transfer from another facility, wound location (anterior/posterior), trauma team activation, day versus night presentation (7 pm vs. 7 am), body mass index (BMI), Charlson Comorbidity Index (CCI),^14^ and Injury Severity Score (ISS). Age and BMI were highly collinear with other variables as assessed by variance inflation factor (12.8 and 7.8, respectively) and were excluded from the model.

Multivariable logistic regression and Pearson χ^2^ test were used to assess predictive variables as appropriate. A significance threshold of *P* < 0.05 was used. All analysis was conducted using statistical software (JMP version 15. SAS Institute Inc, Cary, NC, 1989–2021).

## 3. Results

**FIGURE 1.**
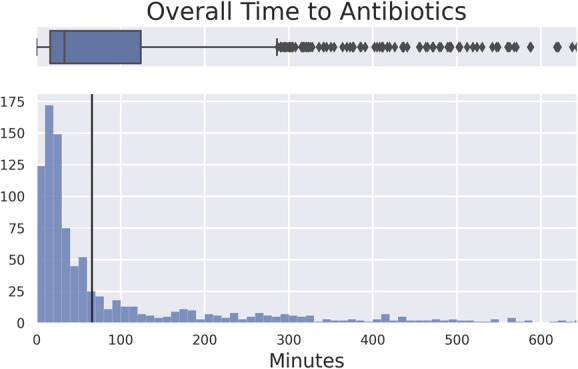
Time from ER arrival to administration of antibiotics in minutes. Vertical black line represents 66-minute threshold for overall delay (top fifth percentile of data points omitted for readability).

Nine hundred sixty-three records of patients with open fractures were identified, with a median time to antibiotic administration of 33 minutes and an interquartile range of 108 minutes (Fig. 1). Isolated injury (*P* = 0.046; OR 1.434; 95% CI 1.007–2.043), Charlson Comorbidity Index (*P* = 0.046; OR 1.434; 95% CI 1.007–2.043), and transfer from another facility (*P* < 0.001; OR 4.555; 95% CI 3.076–6.745) were associated with overall delay (>66 minutes; Table 1). Sex, wound location, trauma team activation, time of presentation, and ISS did not significantly correlate with time to antibiotic administration (*P* > 0.05).

**TABLE 1:** Multivariable Logistic Regression Model for 66-Minute Overall Delay

	Odds Ratio	95% Confidence Interval	*P*
Sex (female)	1.364	0.999–1.862	0.051
Isolated fracture*	1.434	1.007–2.043	**0.046**
Day team	0.804	0.597–1.083	0.152
Anterior wound	1.034	0.652–1.642	0.887
Transfer*	4.555	3.076–6.745	**<0.0001**
Charlson Comorbidity Index*	0.900	0.826–0.982	**0.018**
Injury Severity Score	1.004	0.984–1.025	0.688
Trauma team activation	1.033	0.692–1.542	0.874

* Significant by multivariable logistic regression.

Bold indicates *P* < 0.05 significance.

Three hundred sixty-four patients were administered antibiotic before entry of the order into the EMR and were excluded from analysis of pharmacy delay. One hundred thirty-eight patients had a delay in pharmacy/nursing preparation, defined as delay in administration after initial medication order placed, greater than 1 interquartile range above the median (greater than 78.4 minutes). This delay was associated with trauma team activation (*P* < 0.0001; OR 5.383; 95% CI 2.916–9.939), transfer from another facility (*P* = 0.0004; OR 2.416; 95% CI 1.479–3.946), and higher Injury Severity Score (*P* = 0.0015; unit OR 1.039; 95% CI 1.007–1.069; Table 2). Nonisolated injury, sex, wound location, CCI, and time of presentation did not significantly correlate with significant delay in pharmacy or nursing preparation (*P* > 0.05).

**TABLE 2:** Multivariable Logistic Regression Model for Pharmacy Delay of Antibiotic Administration

	Odds Ratio	95% Confidence Interval	*P*
Sex (female)	1.158	0.732–1.833	0.531
Isolated fracture	0.992	0.596–1.653	0.976
Day team	0.673	0.428–1.058	0.087
Anterior wound	0.837	0.414–1.693	0.62
Transfer*	2.416	1.479–3.946	**0.004**
Charlson Comorbidity Index	0.911	0.802–1.035	0.151
Injury Severity Score*	1.038	1.007–1.069	**0.015**
Trauma team activation*	5.383	2.916–9.939	**< 0.0001**

* Significant by multivariable logistic regression.

Bold indicates *P* < 0.05 significance.

## 4. Discussion

The purpose of this study was to determine which patient-specific and systemic factors are associated with a delay of antibiotic administration after a patient arrives at the hospital. Wound location did not seem to be associated with a delay, but isolated injury did correlate with increased overall delays. Isolated, simpler-appearing fractures may be formally and informally triaged as less acute, thereby increasing the time before a physician assesses the patient and thus diagnoses an open fracture. Similarly, higher CCI is inversely correlated with overall delay—we infer that patients presenting with medical comorbidities are more likely to be prioritized for evaluation and to receive more timely care.

Delays were also more likely among patients who had been transferred from a referring health care facility. Patient transfer inevitably introduces additional complexity into the medical decision-making process. Patient transfers to this institution are typically preceded by interfacility communication, with the explicit purpose of facilitating smooth and expeditious care. Consequently, one might expect that antibiotics would be more quickly administered to these patients after their arrival to the ED. The quality of this communication is often limited; however, orthopaedic physicians are not often directly involved. A failure to transfer all relevant information or the exclusion of key agents may therefore delay recognition of open fractures and diffuse responsibility for providing antibiotic prophylaxis. Moreover, transfer patients often present with limbs splinted and wounds obscured. Anecdotal experience suggests that the receiving physicians may be less comfortable taking down splints in the emergency department to expose extremity injury sites when compared with orthopaedic consultants, marginally delaying assessment further.

It should be noted that the effect of transfer on overall time to antibiotics may be exaggerated by the inclusion of patients to whom antibiotics were, in fact, administered before arrival. Although patients were excluded from analysis if the medical record noted the administration of appropriate antibiotics, some patients may have nonetheless received antibiotics appropriately before transfer, likely by the transferring facility, with this knowledge passed to ED staff but without documentation in the medical record. Any such patient then given additional doses of antibiotics at an appropriate interval would have been captured in our analysis as a delayed administration. While this suggests some caution in interpretation of our results regarding transfers, the number of these cases is believed to be relatively low and thus should not nullify evidence shown here that transfers carry risk of delay.

A surprising 14.3% of our patients had a delay after order placement which we prespecified as median time between order an administration plus one interquartile range. We termed this as a pharmacy delay, although we recognize that this is likely multifactorial. All patients who met our criteria for pharmacy delay would also experience overall delay in antibiotic administration. We found that transfer from another health care facility was significantly associated with pharmacy-related delay because it was associated with overall delay, underlining the importance of targeting these patients in future intervention. Although trauma team activation and Injury Severity Score are not associated with overall delays, they are both associated with increased pharmacy-related delay and the effect of trauma team activation is particularly strong (OR 5.4). This may be because trauma activation introduces multiple teams to comanage the patient, or potentially lifesaving, prioritized interventions.

This study identified multiple modifiable and nonmodifiable risk factors for delayed administration of antibiotics, thereby providing targets for future quality improvement efforts. Preparing antibiotics, stocking them in the emergency room, and accepting a lower treatment threshold for their administration may significantly reduce the delays detected by our analysis by preempting the time required for medical decision making and drug preparation. This concords with findings from Shieh et al^15^ who recently found that formalizing an antibiotic prophylaxis protocol, identifying a single member of the medical team responsible for placing prophylaxis orders, and regularly auditing performance were sufficient to reduce antibiotic administration times. Interestingly, we did observe a trend in day time presentation being protective (OR 0.673, 95% CI 0.428–1.058, *P* = 0.087) of delay, which we would expect given well-known benefits to efficiency with staff more familiar with protocolized treatments.^16–18^

Our results complement the body of literature highlighting the virtues of prearrival antibiotics administered by emergency medical services. Multiple sources have described protocols of prehospital interventions for patients with various conditions, and recently, Lack et al 2019 reported the successful implementation of prophylactic antibiotic administration by EMS in cases of suspected open fractures, without increases in adverse reactions.^19–22^ Systemic changes to implement antibiotic administration at transferring facilities or by transferring EMS may be similarly successful and worthy of trial. Neither our trauma center nor the regional EMS system has a standardized protocol of prehospital antibiotic prophylaxis, but the premise of prestocking antibiotics and administering them before a full evaluation by a physician may achieve the same result.

To address some of the findings of the presented study, some changes were made at our institution to improve any delay. To address deficiencies in transfers and isolated injuries, education was given to the emergency room staff, namely in removal of splints and/or prompt consultation with orthopaedics to evaluate the patient with known/suspected injury. To improve delays associated with the pharmacy, we have brought premixed antibiotics (including weight-based gentamicin) to our trauma bays to allow for more prompt administration. Data are still being gathered on the effectiveness of these interventions.

Some of the weaknesses of our study include the retrospective nature and inaccuracies of the medical record. Similarly, the generalizability of our results should be considered with the understanding that patient-specific and in-hospital factors contributing to delays are likely to be highly variable between sites, with urban/regional facilities having differing prehospital conditions/transport times. This study was conducted at a large academic institution with a significant rural catchment, a high proportion of transferred patients, and an accordingly high variability of prehospital times and protocols. As a result, we have likely captured a “worst case” scenario which might be most revealing. Future studies may aggregate data from a variety of centers to increase the generalizability of their conclusions.

## 5. Conclusions

Prompt antibiotic administration after open fractures is important to preventing infection. This study demonstrated that transfer from an outside facility, isolated injuries, and the underlying health status of the patient can contribute to delay. Education and modification of protocols may help prevent these delays. Knowledge gained from this investigation may help guide others to modify modifying existing and creating new protocols to address some of these factors to ultimately expedite antibiotic administration and thereby reduce the incidence of postfracture infection.
